# The In Vitro Antimicrobial Susceptibility of *Borrelia burgdorferi* sensu lato: Shedding Light on the Known Unknowns

**DOI:** 10.3390/pathogens12101204

**Published:** 2023-09-28

**Authors:** Klaus-Peter Hunfeld, Peter Kraiczy, Douglas E. Norris, Benedikt Lohr

**Affiliations:** 1 Institute for Laboratory Medicine, Microbiology & Infection Control, Northwest Medical Centre, Academic Teaching Hospital, Medical Faculty, Goethe University Frankfurt, Steinbacher Hohl 2-26, D-60488 Frankfurt am Main, Germany; lohr.benedikt@khnw.de; 2 INSTAND e.V., Gesellschaft zur Förderung der Qualitätssicherung in medizinischen Laboratorien e.V., Ubierstraße 20, D-40223 Düsseldorf, Germany; 3 Institute for Medical Microbiology & Infection Control, University Hospital Frankfurt, Goethe University Frankfurt, Paul-Ehrlich Str. 40, D-60596 Frankfurt am Main, Germany; kraiczy@em.uni-frankfurt.de; 4 W. Harry Feinstone Department of Molecular Microbiology & Immunology, Bloomberg School of Public Health, Johns Hopkins University, 615 N Wolfe St, Baltimore, MD 21205, USA; douglas.norris@jhu.edu

**Keywords:** antimicrobial agents, antimicrobial resistance, *Borrelia burgdorferi*, in vitro persistence, in vitro susceptibility, spirochetes, susceptibility testing

## Abstract

Human Lyme borreliosis (LB) represents a multisystem disorder that can progress in stages. The causative agents are transmitted by hard ticks of the *Ixodes ricinus* complex that have been infected with the spirochete *Borrelia burgdorferi* sensu lato. Today, LB is considered the most important human tick-borne illness in the Northern Hemisphere. The causative agent was identified and successfully isolated in 1982 and, shortly thereafter, antibiotic treatment was found to be safe and efficacious. Since then, various in vitro studies have been conducted in order to improve our knowledge of the activity of antimicrobial agents against *B. burgdorferi* s. l. The full spectrum of in vitro antibiotic susceptibility has still not been defined for some of the more recently developed compounds. Moreover, our current understanding of the in vitro interactions between *B. burgdorferi* s. l. and antimicrobial agents, and their possible mechanisms of resistance remains very limited and is largely based on in vitro susceptibility experiments on only a few isolates of *Borrelia*. Even less is known about the possible mechanisms of the in vitro persistence of spirochetes exposed to antimicrobial agents in the presence of human and animal cell lines. Only a relatively small number of laboratory studies and cell culture experiments have been conducted. This review summarizes what is and what is not known about the in vitro susceptibility of *B. burgdorferi* s. l. It aims to shed light on the known unknowns that continue to fuel current debates on possible treatment resistance and mechanisms of persistence of Lyme disease spirochetes in the presence of antimicrobial agents.

## 1. Introduction

Human Lyme borreliosis (LB) represents a multisystem disorder that can progress in stages. It is transmitted by ticks of the *Ixodes (I.) ricinus* complex that have been infected with the spirochete *Borrelia burgdorferi* sensu lato [1]. The causative agent of LB remained a mystery until the discovery of spirochetes in the midgut of ticks collected on Long Island, New York in 1982 by the Swiss-borne entomologist Willy Burgdorfer [2]. Subsequent epidemiological and laboratory investigations led to one of the most important biomedical discoveries of the 20th century: the establishment of LB as a new infectious disease entity [3]. Today, LB is regarded as the most important human tick-borne illness in the northern hemisphere [4]. Annual incidence rates in Europe range from 0.001/100,000 in Italy (2001–2005) to 111/100,000 in Germany and 188.7/100,000 in Slovenia (2014) [5,6,7,8,9]. According to the Centres for Disease Control and Prevention (CDC), the incidence in the United States of America (U.S.A.) in 2014 was 7.9/100,000, with the majority of cases reported in the northeastern and upper midwestern states [10]. Recent modelling studies based on claims data, however, suggest significant under-reporting and predict much higher annual incidence numbers for LB of >300,000 (93/100,000) in the U.S.A. and >200,000 (260/100,000) in Germany [10,11]. Antibiotic treatment was shown to be successful in empirical therapeutic trials soon after the causative agent was correctly identified and successfully isolated in 1982 [12,13,14]. Subsequently, a variety of in vitro and in vivo studies have been conducted to further characterize the activity of antimicrobial agents against *B. burgdorferi* s.l., and to determine the clinically most efficacious stage-dependent antibiotic therapy for LB [15,16,17]. Culture-proven treatment failures are rare but have been reported in LB patients for almost every suitable antimicrobial agent [7,16,18,19,20,21,22]. However, the current understanding of the persistence or possible mechanisms of resistance in *B. burgdorferi* s.l. remains limited and is largely based on in vitro experiments performed on a relatively few isolates. Moreover, deeper insights into the interactions between antimicrobial agents and the pathogen, and into the possible phenotypic or genotypic mechanisms of resistance, as gained from animal models and in vitro experiments, are sparse. The exact mechanisms the spirochetes use to survive in LB patients with culture-proven treatment failure are not known [7]. This review summarizes what is and what is not known about in vitro susceptibility testing methods and about the in vitro interactions of *B. burgdorferi* s.l. with antimicrobial agents and antibiotic medium preparations. In addition, open questions and current limits of knowledge surrounding possible mechanisms of persistence and antimicrobial resistance of borreliae will be addressed which continue to fuel the ongoing debate about the possible persistence of these spirochetes following exposure to antimicrobial agents.

## 2. The Causative Agents

**Figure 1:**
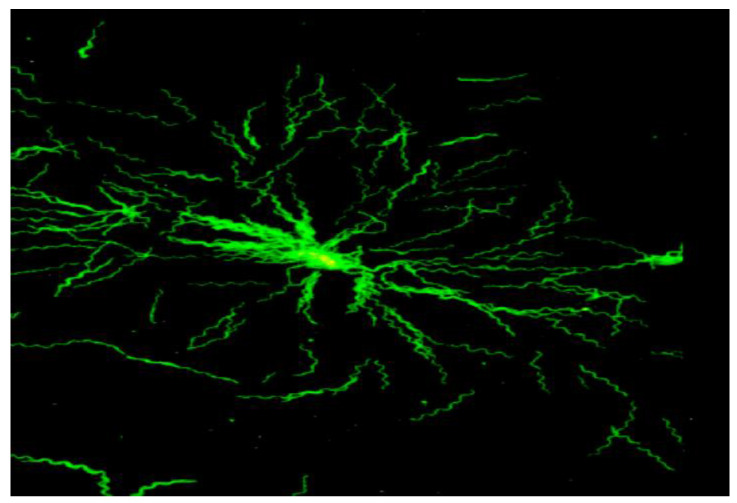
Microcolony of *B. garinii*, Immunofluorescent staining, oil, 1000× (modified from [32]).

The spirochetes that cause LB belong to the so-called *B. burgdorferi* s.l.-complex. They are spiral-shaped bacteria (Figure 1) of 4–30 µm in length and 0.2–0.3 µm in diameter. They are members of the Spirochaetaceae family and belong to the genus *Borrelia* which comprises both the relapsing fever borreliae and the closely related LB agents. *Borrelia burgdorferi* s.l. is transmitted by members of the *Ixodes ricinus* complex. These are predominantly *I. ricinus* and *I. persulcatus* in Europe and Asia, and *I. scapularis* and *I. pacificus* in North America [23]. Once infected with borreliae, these three-host hard ticks (Ixodidae) can remain infected for the remainder of their lives, including through molts. Thus, they are able to effectively transmit spirochetes in the next feeding stage and potentially to their hosts [24]. The geographical presence of the disease in the northern hemisphere mirrors the distribution of the *Ixodes* spp. ticks that transmit the LB agents. At present, the *B. burgdorferi* s.l.-complex includes 23 different genospecies. However, only *B. burgdorferi* sensu strictu, *B. afzelii*, *B. spielmanii*, *B. garinii,* and *B. bavariensis* have been definitively established to be pathogenic to humans [25,26,27,28]. Very recently, *B. bissettiae* and *B. mayonii* have been described as causes of LB in clinically ill patients from Germany, the U.S.A. and Canada [29,30,31]. Human pathogenicity is also very probable but remains a matter of debate for *B. valaisiana* [27] and *B. lusitaniae* [28]. All of the above-mentioned species that are assumed to be pathogenic to humans, with the exception of *B. mayonii,* are found in Europe. *Borrelia burgdorferi* s.s., *B. mayonii* and the probable pathogen *B. bissettiae* are present in the U.S.A., and all species listed here are distributed in Asia, except for *B. burgdorferi* s.s. and *B. mayonii* [26].

## 3. Clinical Manifestations

There are often no clinical signs or symptoms immediately after a tick bite. However, once clinically apparent, the disease can manifest as a multisystem disorder for about 5 to 10% of individuals, exhibiting a wide variety of clinical symptoms [1,33]. Typically, a site-specific infection occurs following pathogen multiplication at the site of the infective tick bite. In clinically apparent cases it is then associated with early, localized disease (erythema migrans, EM) [33]. Bacteraemia with nonspecific symptoms, such as fever and malaise, can occur in some individuals. Survival of borreliae in blood requires complement evasion in the host. Recent evidence suggests that proteins like CspZ, a newly recognized spirochete surface protein, facilitate resistance to complement-mediated killing in vitro by binding to the complement regulator factor H and thereby promoting a systemic infection in vertebrate hosts [34]. Hematogenous dissemination is then followed by typical early disseminated disease manifestations, such as multiple EM, neurological manifestations (e.g. poly-meningo-radiculoneuritis, also known as Bannwarth syndrome, facial palsy), and possibly late manifestations including Lyme arthritis (LA), and acrodermatitis chronica athrophicans (ACA) [33]. The various genospecies of the *B. burgdorferi* s.l.-complex are genetically very heterogenous [25]. Despite the fact that all human pathogenic genospecies can cause EM, some genospecies are associated with distinct symptoms. For example, *B. afzelii* is the predominant genospecies in patients with ACA and causes the majority of EM cases in Europe. *B. garinii* and *B. bavariensis* are often associated with neurological manifestations in Europe and, *B. burgdorferi* s.s. is primarily associated with LA and manifestations affecting the joints in Europe and the U.S.A, and almost exclusively is the cause for EM in the U.S.A [31]. *B. spielmanii* has so far only been isolated from EM in Europe [25,35] and the organ tropisms of *B. mayonii* and *B. bissettiae* are not yet firmly delineated.

## 4. In Vitro Susceptibility Testing Methods for *B. burgdorferi* s.l.

Currently, there are many types of in vitro susceptibility testing for *B. burgdorferi* s.l.; however, the methods vary widely. Over the years, various modifications have been made to the micro- and macrodilution methods, and different inocula, and media (i.e. Barbour-Stoenner–Kelly [BSK] medium, modified Kelly-Pettenkofer [MKP] medium) have been used for testing [36,37,38,39,40,41,42,43,44,45,46,47,48,49,50]. For the most part, samples were checked after antibiotic exposure for the presence of spirochetes using dark-field microscopy or by macroscopic evaluation of microtitre vials. However, the enumeration of motile spirochetes and visual confirmation of discrete morphological changes in microtitre plates is time-consuming, difficult to standardize, and often provides no easily measurable minimal inhibitory concentrations (MIC) endpoints [17,49,51].

### 4.1. Determination of Minimal Inhibitory Concentrations (MIC)

Dever et al. [40] developed a relatively reproducible susceptibility test based on broth microdilution with BSK medium, an initial inoculum size of ~10^6^ bacterial cells per mL, and an incubation time of 72 h. Wells are assessed visually for growth. The MIC is usually defined as the lowest concentration of an antimicrobial agent where no presence of a sediment on the bottom of the wells and no discrete color change in the BSK medium can be observed with the naked eye.

### 4.2. Microdilution in Combination with Microscopic MIC Determination

A number of modified microdilution susceptibility testing methods using BSK medium have been developed over the years [52,53]. In some tests, MIC values are measured by exposing an inoculum with a final density of ~10^5^ borreliae/mL, as determined in a Neubauer counting chamber, to logarithmic dilutions of antimicrobial agents. Sterile 96-well microtitre plates with the inclusion of positive and negative growth controls are used. The plates are then inoculated under sterile conditions before being sealed with adhesive plastic and incubated at 33 °C under anaerobic conditions for 72 h. Following incubation, dark-field microscopy is used to examine all wells for spirochete counts, morphology and motility [36,37,40,41,43,44,51]. Here, the MIC is commonly defined as the lowest concentration of an antimicrobial agent at which no motile or only very slightly motile spirochetes are observed in significantly reduced numbers using dark-field microscopy. Antimicrobial agents are normally tested in triplicate. MIC values, in which 50% (MIC_50_) and 90% (MIC_90_) of isolates are inhibited, are determined for each antibiotic used on the isolates tested. However, such experiments are laborious and MIC determination can be very imprecise as it highly depends on the individual investigator. Unfortunately, no internationally accepted standardized MIC breakpoints for antimicrobial agents currently exist for *Borrelia* spp. [53,54].

### 4.3. Microdilution in Combination with Colorimetric MIC Determination

**Figure 2:**
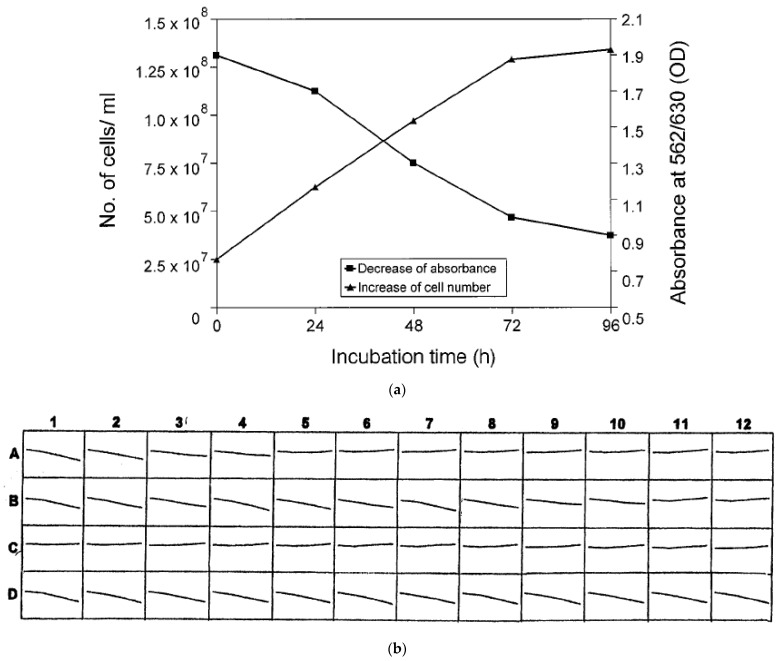
(**a**). Sensitivity of the MIC method as determined for *B. burgdorferi* s.s. reference strain B31 by investigation of growth using conventional cell counts and colorimetric examinations. Experiments were performed in duplicate on different days (modified from [51]). (**b**). Kinetic measurement of MIC as performed by software-assisted colorimetric microdilution method for reference strain B31 (modified from [51]). Rows: A, tetracycline (0.015–32 mg/L); B, amikacin (0.06–128 mg/L); C, piperacillin (0.06–128 m/L); D, growth control. Growth of samples and controls was determined for each well based on the decrease of absorbance (A562:630) after 72 h (E_t72_) in comparison to the initial absorbance values (E_to_). The lowest concentration at which no or a minor color shift (decline of the curve) could be detected in comparison to the growth control was interpreted as the MIC: Tetracycline: row A, well no. 5 (MIC: 0.25 mg/L); amikacin: row B, well no. 11 (MIC: 64 mg/L); piperacillin: row C, well no. 1 (MIC: 0.06 mg/L).

As outlined above, early antimicrobial susceptibility testing of *B. burgdorferi* s.l. resulted in variable MIC values due to the lack of a standardized method and issues with endpoint determination. As a result, a colorimetric microdilution assay using modified BSK was developed as an alternative to microscopic MIC determination. Here the general principles and practices of conventional antimicrobial susceptibility testing that are established for other microorganisms are used to analyze the activity of antimicrobial agents against *B. burgdorferi* s.l. isolates [51,55]. The method has also been used to investigate in vitro antimicrobial resistance patterns of *B. spielmanii* and *B. valaisiana* strains as well as a *B. bissettiae* tick isolate [55,56]. The test system is based on color changes that occur in BSK medium when phenol red as a pH indicator is added. The color change indicates an accumulation of non-volatile acids produced by actively metabolizing spirochetes. Final concentrations of the lyophilized antibiotics of the preloaded microtiter plates are reconstituted by adding 200 µL of the final inoculum suspension (5 × 10^6^ cells) in BSK medium containing phenol red (25 mg/mL) as the growth indicator. The same batch of BSK medium derived from commercially available or self-composed components should be used throughout the experiments [51,55]. It is very important that the pH be adjusted to ~7.6 before testing. Microtitre plates containing spirochetes and growth controls must then be immediately sealed with sterile adhesive plastic and cultured at 33 °C with 5% CO_2_. The presence or absence of growth is then examined after 0, 24, 48, and 72 h by kinetic measurement of the indicator color shift at 562/630 nm using a commercially available reader (e.g. PowerWave 200; Bio-Tec Instruments, Winooski, VT, U.S.A.) in combination with a software-assisted calculation program (e.g. Microwin 3.0; Microtek, Germany; Figure 2a,b) [51,55].

Finally, growth in the samples and controls is determined for each well based on a decrease in absorbance after 72 h (E_t72_) over the initial absorbance values (E_t0_) by performing a software-assisted calculation of the growth curves (Figure 2). A determination is made mathematically as follows: If, at 72 h, the absorbance values have decreased by 10% or more over the initial absorbance values, the well is considered positive for growth of borreliae (E_t72_ ~ E_t0_ minus 10%). In order to determine the endpoints for each isolate tested, the growth characteristics of the test and control wells are compared. The lowest concentration of antibiotic for which no significant color shift can be detected is considered the MIC. It has been established that the growth of spirochetes in a microtitre well results in a 10% decrease in absorbance when compared to the initial absorbance values of the microtitre well after 72 h [51,55,56,57,58]. Recently, a slightly modified assay also proved suitable for testing relapsing fever spirochetes, including *B. hermsii* and *B. miyamotoi* [48,49]. However, MKP medium was used instead of BSK medium and there have been reported challenges with variation of initial absorbance values throughout the experiments as well as with detecting the smaller decrease in absorbance compared to the initial absorbance values in some *Borrelia* strains with low replication rates [48,49]. As outlined above, this can happen due to the variability of the serum batches used for the medium preparation and/or inconsistencies in the timely preparation and transfer of the medium to the test plates, as well as the lack of a precise pH-adjustment before the experiment. Consequently, in this study, an alternative MIC calculation has been suggested. Here, the colorimetric MIC is calculated by comparing the drop in absorbance (Et_0_–Et_72_) to the drop in absorbance of the positive control (no antibiotics) (E_POS,t0_–E_POS,t72_). The MIC is set arbitrarily at 25% [48,49]. Thus, in this study, the colorimetric MIC was defined as the lowest concentration of antibiotics where (E_t0_–E_t72_) is 25% of (E_POS,t0_–E_POS,t72_). The authors suggest the use of this formula as a more robust alternative when calculating colorimetric MIC values but fail to demonstrate clinically significant differences between the colorimetric MIC values as calculated by the two alternative approaches and by dark-field microscopy [48,49]. Also, no reason is given for the arbitrarily chosen threshold, and no ATCC reference strains were used in these investigations to control and adjust for possible negative interference of antibiotics, test medium or test methodology. Bearing in mind also the small number of strains and substances tested, significant differences in these two approaches are not apparent and we would strongly suggest the use of the original method using modified BSK medium for experienced laboratories.

In summary, colorimetric microdilution testing is well-established as the testing method of choice for borreliae and offers the benefits of reliability, reproducibility, convenience and standardization. Moreover, it can handle large numbers of isolates and antibiotics under standardized conditions. It is important to note, though, that, in addition to a timely and accurate preparation of the BSK medium, antibiotics and inoculation of the plates, the pH of the final medium must be carefully adjusted in order to achieve comparable results. Moreover, for purposes of quality control, reference strains, such as *Staphylococcus aureus* ATCC 29213, *Escherichia coli* ATCC 25922, and *Pseudomonas aeruginosa* ATCC 27853, should be examined with the same assay, in the same medium and under the same test conditions after 24 h of incubation according to NCCLS guidelines [51,55,56,57,58,59].

### 4.4. Approaches for MBC Determination Using Macro- and Microdilution Methods

*Borrelia burgdorferi* s.l. is a fastidious organism that can evade the immune system. Its persistence in the host can lead to late-stage manifestations. Similarly, the survival of a small number of bacteria may result in persistence or a clinical relapse [7,60,61,62,63]. This is why, in contrast to testing of common rapid growers, there is good reason to apply more stringent test conditions and longer periods of subculture when investigating the borreliacidal capacity of widely used antimicrobial agents so as to understand the possible regrowth of the pathogen after exposure to the antibiotic [17,52]. Moreover, it appears that most antimicrobial agents need a longer length of time to kill *B. burgdorferi* s.l., even when compared to other spirochetal infections like *Treponema pallidum* [17,52,64]. Likewise, it must be taken into account, that a 3 log reduction (99.9%), as commonly used to define the MBC in rapid growers, also means the survival of 10 to 10^4^ bacteria per mL at two to four log_2_ unit dilutions above the MIC, depending on the final inoculum (10^4^–10^7^ per mL) and the substance being tested [51,55].

Many studies on the in vitro susceptibility of borreliae, therefore, tend to use variations of micro- and macrodilution methods; however, the MBC criteria and the subculture periods for the detection of possible re­growth of the antibiotically treated borreliae tend to vary widely (seven days to three weeks) (Table 1). Usually, MBC values are determined after 72 h of incubation with the antibiotic by taking fresh aliquots from all vials lacking detectable growth and diluting them at a ratio of 1:75 with fresh BSK to achieve a sample dilution below the MIC. Incubation then continues at 33 °C in 5% CO_2_ for an additional 3 weeks [17,52] to assess the drug concentrations that provide a 100% killing of the initial inoculum under stringent conditions. The MBC is therefore commonly defined as the lowest concentration of the antimicrobial agent where no spirochetes can be detected after 5 to 10 high-power fields are examined by dark-field microscopy for the presence or absence of spirochetes after 3 weeks of subculture, i.e., 100% killing under rigorous conditions [52,55,65]. The efficacy of the tested antimicrobial substances in killing 100% of the inoculated microorganisms after 72 h is thus a very stringent criterion for any antibiotic and, as outlined above, leads to higher MBC values than those usually obtained by conventional time-kill studies and investigations using less restrictive MBC definitions. Such an approach also clearly differs from the traditional testing methodology commonly used in non-fastidious microorganisms but can be helpful in identifying substances that are more appropriate for the antimicrobial chemotherapy of LB [17,52].

### 4.5. Time-Kill Experiments Using Macro- and Microdilution Methods

**Figure 3:**
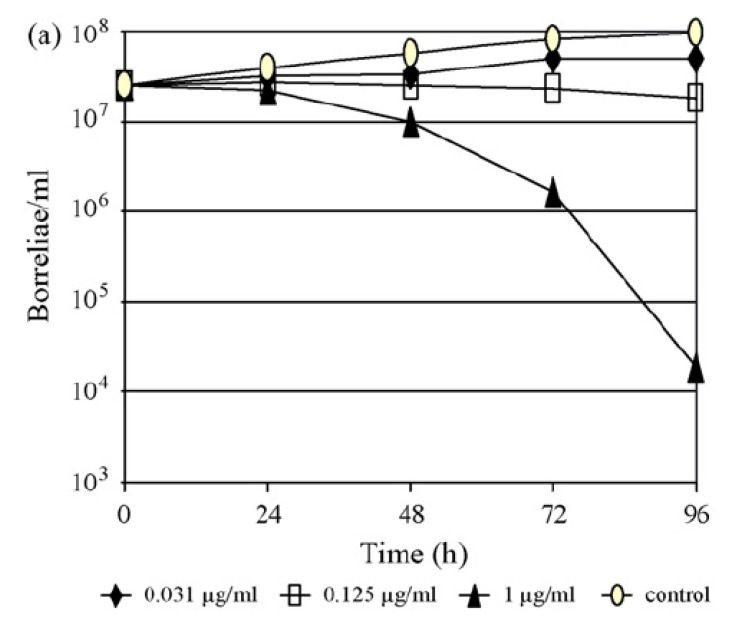

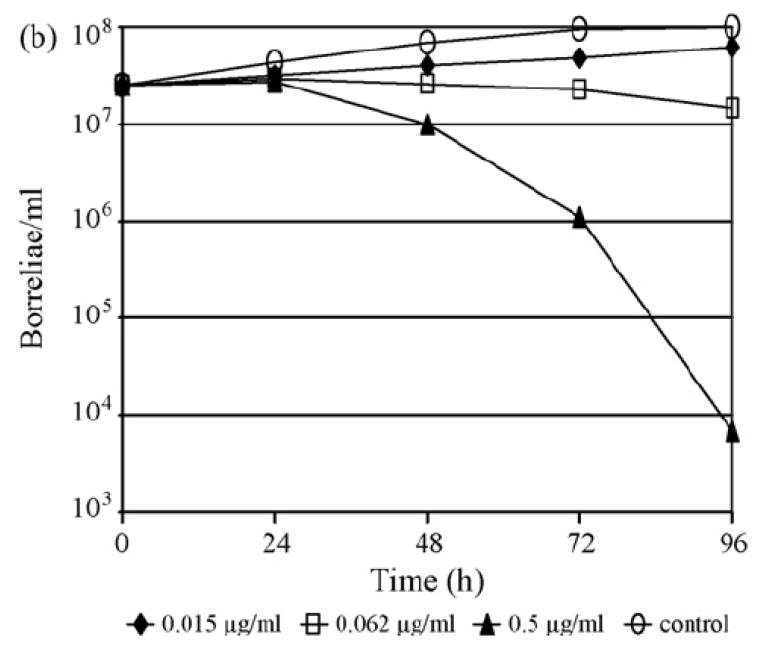
(**a**,**b**). Time–kill curves for *B. afzelii* FEM1 (adapted from [77]) with (**a**) ertapenem and (**b**) ceftriaxone at 0.25× the minimal inhibitory concentration (MIC), at the MIC and at three log2 unit dilutions above the MIC. Experiments were performed on different days by investigation of growth using conventional cell counts and data were reported as the means of two experiments.

Time-kill curve techniques that demonstrate a 99.9% kill of the tested organism correlate best with cure rates and clinical outcomes as demonstrated for rapid growers in animal models [73,74]. In in vitro assays involving *B. burgdorferi* s.l., the kinetics of killing are usually assessed by determining the percent viability of intact motile spirochetes using dark-field microscopy (Figure 3) following antibiotic exposure in a liquid medium [17,52,64,75,76].Even though borreliae are sensitive to relatively small concentrations of penicillin and ceftriaxone, they are known to die very slowly [64] (Figure 3). Experiments are usually performed on different days, starting with variations of micro- and macrodilution methods in combination with dark-field microscopy. Spirochete counts are then reported as the mean of at least two experiments. As motility strongly corresponds to the viability of the spirochetes [75,76], samples and controls (200 μL) of the final inoculum suspension (commonly 5 × 10^6^ borreliae/well) for each isolate are then investigated for morphologically unaffected motile borreliae by conventional cell counts [17,52]. Cell counts are usually performed on different days for each of the isolates tested after 0, 48, 72, 96, and 120 h of incubation by applying identical test conditions to samples (in the presence of) and controls (in the absence of) antibiotics. A substance well known for its high activity against borreliae (such as ceftriaxone) and a substance with low activity (such as an aminoglycoside) are usually also applied as technical controls. *Borrelia* isolates are then used to determine both generation times in BSK medium and rates of killing on the part of the tested antimicrobial agents. Concentrations at four times the respective MIC values are typically used [17,52,57].

### 4.6. Time-Kill Experiments Using the Subsurface Plating Technique (SPT)

Some authors have also utilized solid media through the application of a subsurface plating technique (SPT) as a way to determine more sophisticatedly a 99.9% killing rate through colony counting [40]. Nonetheless, comparative studies on the suitability of solid media for the successful culture of borreliae showed considerable variation in the recovery rate of the pathogen after two weeks of subculture [78].The applicability of using solid media to determine borreliacidal activity of antimicrobials may also be limited owing to the fact that some isolates are incapable of growing on a solid medium within a reasonable period of time [55,78]. Conventional time­kill experiments in combination with SPT are laborious, insufficiently standardized, and most importantly, impractical for testing larger numbers of *Borrelia* strains. In addition, the technique has been used mainly for fast growing *B. burgdorferi* s.s. and has yet to be evaluated for its suitability to test isolates belonging to different genospecies and slow-growing strains.

### 4.7. Approaches for MIC Determination Using a Dialyses Culture Method

The continuous decline in drug concentrations over time has been identified for some agents such as penicillin and cephalosporins in BSK medium. To circumvent such interactions, some authors propose the use of a dialysis culture method to determine the MIC and MBC values of penicillin more accurately for borreliae [71,79]. In this method, enclosed bacterial suspensions are sealed in dialysis membrane bags and are then transferred daily to new tubes containing BSK medium with freshly added antibiotics for an overall incubation period of six to seven days. No comparable method for the susceptibility testing of other microorganisms exists where cultures are replenished with fresh medium-antibiotic preparations daily [80]. Therefore, a major drawback of the dialyses culture method is that it is difficult to standardize, and the MIC values obtained cannot be compared with those of other studies or of other bacterial pathogens [53].

## 5. In Vitro Antimicrobial Susceptibility Pattern of the *B. burgdorferi* Complex

### 5.1. Spectrum of In Vitro Susceptibility against Antimicrobial Agents

Even though several antimicrobial agents have been tested for their in vitro activity against borreliae, the full spectrum of antibiotic susceptibility has yet to be defined. Furthermore, the resistance pattern of *B. burgdorferi* s.l. is difficult to predict as it clearly differs from that of common Gram-negative bacteria (Table 2) [17,52]. To further expand our knowledge of the in vitro susceptibility profile of the pathogen, attempts in recent years have been made to explore currently available antimicrobial agents against a larger number of borreliae isolates in order to better characterize the in vitro susceptibility of *B. burgdorferi* s.l. [17,52,57].

### 5.2. ß-Lactam Agents

ß-lactams belonging to the penicillin class of antimicrobial agents, such as penicillin, amoxicillin, and III generation cephalosporins such as ceftriaxone, have been shown to be highly active against *B. burgdorferi* s.l. in vitro. They are also clinically efficacious and, thus, regarded as agents of choice when it comes to the treatment of LB [52,55,65]. The penicillin derivatives mezlocillin, azlocillin and piperacillin seem to be even more active than penicillin and amoxicillin (Table 2), whereas sulbactam and the monobactam aztreonam revealed no significant in vitro activity against *B. burgdorferi* s.l. [58].

In terms of the cephalosporins, the group II compound cefuroxime is highly active against borreliae, whereas loracarbef is not [81]. Ceftriaxone, cefotaxime, cefdinir and cefixime are effectual, while other group III agents like ceftamet-pivoxil, ceftibuten and cefpodixime-proxetil are inefficacious in vitro. From this, it is clear that the activity of compounds against *B. burgdorferi* s.l. belonging to a specific class of substances (e.g., cephalosporins) does not seem to correspond to the traditional grouping of these antimicrobial agents with regard to their spectrum of activity against Gram-negative and Gram-positive bacteria (Table 2). Interestingly, these observations resemble the findings for the activity of cephalosporins against *Leptospira*, which also show variable activity independent of the cephalosporin groups [81].

With respect to the carbapenems, the testing of 11 isolates of *B. burgdorferi* s.l. against faropenem, ertapenem, imipenem and meropenem demonstrated that ertapenem was the most potent carbapenem on a µg/mL basis, with in vitro activity against borreliae comparable to that of ceftriaxone (Table 2). These findings were supported further by the results of time-kill experiments (Figure 3) in a clinical isolate *of B. afzelii*, demonstrating a >3 log reduction (99.9%) of the initial inoculum after 96 h of exposure to either drug at a concentration of three log_2_ unit dilutions above the respective MIC [17,52,55,65,77].

### 5.3. Macrolides, Azalides and Ketolides

Macrolides are important second-line agents offering treatment options for LB in cases where traditional ß-lactams cannot be administered due to detrimental side effects such as allergies. Current data suggest a rank order of activity for traditional macrolides and azalides against borreliae that corresponds to the efficacy of these agents as revealed by current in vitro susceptibility studies and clinical trials [17,36,50,52,55,61,65,82]. These studies demonstrate higher in vitro efficacy for azithromycin (MIC_90_, 0.0156 μg/mL) than for erythromycin, roxitromycin, or clarithromycin. Median MIC values of the different substances, however, tended to vary over a 10-fold range for individual strains, with the *B. garinii* isolate PSth and the *B. afzelii* isolate EB1 showing the highest MIC values for both the traditional macrolides and the ketolides. In contrast to the findings of Sicklinger et al. [83], other studies found no significant differences in MBC values for the different genospecies tested against macrolides or ketolides, possibly owing to differences in test methodology and inoculum. However, most findings point to inter-strain variability of the in vitro susceptibilities of *B. burgdorferi* s.l. to macrolides rather than to inter-genospecies-specific variations as observed for other antimicrobial agents [53,55,59]. As noted above, testing of *Streptococcus pneumoniae* ATCC 49619 clearly demonstrated increased activity of some macrolides in BSK medium [36,59,84], which might be of consequence for in vitro susceptibility testing of these agents against *B. burgdorferi* s.l. This is critically relevant, for example roxithromycin is highly active in vitro, but is also highly prone to clinical failure when treating patients with LB [61]. This is usually not the case with clarithromycin and azithromycin.

Ketolides are 3-des-cladinosyl-3-oxo 11, 12-cyclic carbamate clarithromycin derivatives and, in contrast to traditional macrolides, exhibit enhanced physiochemical properties and increased antibacterial potency owing to their higher affinity to bacterial ribosomes [85,86,87]. Compounds such as cethromycin and telithromycin belong to this relatively new class of antimicrobial agents that show modifications in the sugar moiety of the lactone ring structure [85,86]. Ketolides have been shown to be highly active against a broad range of aerobic and anaerobic Gram-positive and Gram-negative bacteria, including macrolide-resistant strains [85,87], with an up to 100-fold higher binding affinity to ribosomes than erythromycin [85]. Both cethromycin and telithromycin reduced the number of intact motile borreliae for a more than three log_10_-unit dilutions at 48 to 120 h after incubation at concentrations that were eight times the MIC [59]. Thus, in time-kill experiments, they exhibited a superior in vitro efficacy against borreliae on a µg/mL basis compared to the traditional macrolide derivative erythromycin [59]. However, strain variability was observed and the number of spirochetes tended to decrease more slowly with both substances in the *B. burgdorferi* s.s. isolate PKa-1 [59].

**Figure 4:**
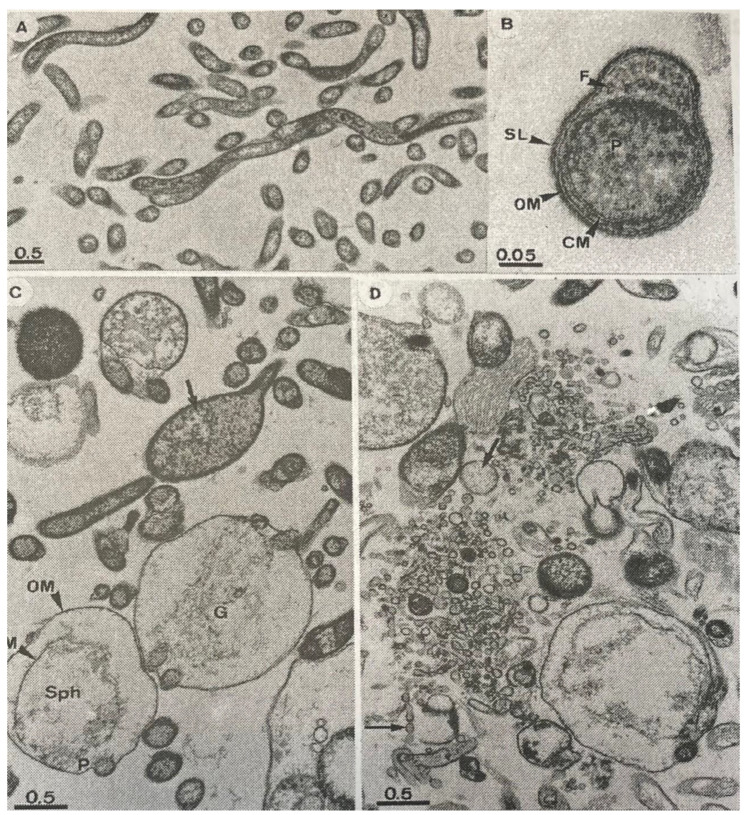
(**A**–**D**): Thin-section electron micrographs of *B. garinii* isolate PSth. (**A**): Control sample at lower magnification without the addition of antibiotics revealing the cell shape and the normal morphology of untreated cells. (**B**): Cross-section of untreated cell at higher magnification showing ultrastructural details of this organism: CM, cytoplasmic membrane; F, flagella; OM, outer-membrane; P, protoplasmic cylinder; SL, surface layer (**C**): Spirochetes exposed to 0.03 µg/mL (15 times the MIC_90_) of cethromycin for 72 h. The OM was often altered or deteriorated, and spheroplast (Sph) formation was observed in earlier stages (e.g., arrow) or in later stages of cell disintegration. Cellular ghosts and ghosts containing cellular debris from the protoplasmic cylinder (G) were observed frequently. (**D**) Spirochetes were exposed to 2 µg/mL (break point concentration for the fastidious organism) of cethromycin for 72 h. The microscopic morphology of the spheroplasts changed drastically; they were further degraded and became fragmented into large numbers of vesicles of different sizes (arrows)—bar length in micrometers in all micrographs.

The excellent in vitro efficacy of cethromycin against borreliae was further substantiated by electron microscope analysis at 4 log_2_ dilutions above the MIC_90_ [0.0312 µg/mL] and at 2 µg/mL, the drug’s tentative breakpoint concentration for fastidious organisms (Figure 4) [87,88]. In terms of the plasma levels of both ketolide agents tested, recent studies have found that achievable maximum plasma concentrations after regular oral dosage are known to be 90 to 270 times higher than the MIC_90_ against borreliae [86,87,89]. Moreover, tissue concentrations exceed the maximum plasma concentrations of both drugs in healthy individuals by tenfold [86,87,89]. According to the results obtained for *S. pneumoniae* strain ATCC 49619 in the quality control experiments of such studies, this remains true even when well-documented antibiotic-medium interactions are taken into account and the obtained MIC values are corrected for one or two log_2_ unit dilutions [52]. Consequently, after further evaluation in clinical studies, ketolides could represent an interesting treatment option in cases of LB where ß-lactams or tetracyclines cannot be administered, or in patients who show resistance to treatment with traditional macrolides [17,53,55,58,59,75].

### 5.4. Tetracyclines and Glycylcyclines

Tigecycline is a primarily bacteriostatic agent belonging to the glycylcycline class of antimicrobial agents, which are modified substances of the tetracycline family. They bind to the 30S subunit of the bacterial ribosome and are known to be very efficacious in vitro against a variety of Gram-negative and Gram-positive bacteria, including multi-drug resistant microorganisms [90,91]. Studies investigating, under standardized conditions, the activity of tigecycline against all genospecies of borreliae isolates in parallel with traditional tetracyclines, such as tetracycline, doxycycline, minocycline and ceftriaxone, as well as cefotaxime [57], revealed the following MIC_90_ rank order: tigecycline > ceftriaxone > cefotaxime > doxycycline > tetracycline. The MBC_90_ rank order was: tigecycline > ceftriaxone > tetracycline > doxycycline > cefotaxime [17,52,55,57,65]. The high in vitro activity of the glycylcycline against borreliae was further substantiated by time-kill experiments performed on *B. afzelii* isolate EB1. Parallel testing of tigecycline and ceftriaxone demonstrated a bacteriostatic effect for 0.016 µg/mL of tigecycline and for 0.03 µg/mL of ceftriaxone after 72 h of incubation. Moreover, tigecycline was bactericidal at a concentration of 0.25 µg/mL, showing a >3 log reduction in the initial inoculum. A concentration of 2 µg/mL was needed for ceftriaxone [17,52,55,57,65]. Taking into consideration the results of the study, the susceptibility testing of mutants with altered tetracycline resistance to tigecycline would be a very interesting future topic for antibiotic research in borreliae [57].

### 5.5. Fluoroquinolones

The use of fluoroquinolones is, in part, well-established for a variety of soft tissue infections [92]. Due to the limited in vitro activity of the initial quinolones against borreliae, in particular nalidixic acid and pefloxacin, fluoroquinolones are not recommended as drugs of choice for the treatment of LB. Some authors report that borreliae shows general resistance to these drugs [93,94]. Other investigators, however, report some in vitro activity [40,58,95,96,97]. Molecular studies on borreliae clearly indicate the presence of a target structure for the quinolones, i.e., a functional DNA gyrase consisting of full-length GyrA and GyrB subunits, which is required for borreliae to grow [98]. Investigations on the activity of 15 fluoroquinolones against human pathogenic isolates of the *B. burgdorferi* s.l. complex and against other previously designated genospecies demonstrated enhanced in vitro activity against borreliae with some of the recently developed antimicrobial agents [58]. Interestingly, the range of MIC values is clearly class dependent, as class I and II compounds, like norfloxacin and ciprofloxacin, generally had higher MIC_50_ and MIC_90_ values than did class III and IV compounds, such as sparfloxacin and gemifloxacin (Table 2). The rank order of potency on a µg/mL basis for the quinolones with enhanced in vitro activity against *B. burgdorferi* s.l. is gemifloxacin > sitafloxacin > grepafloxacin > gatifloxacin, clinafloxacin, trovafloxacin, and sparfloxacin [58].

The higher susceptibilities of *B. burgdorferi* s.l. to class III and IV fluoroquinolones, such as sparfloxacin and gemifloxacin, (Table 2) which are derivatives exhibiting enhanced activity against Gram-positives and anaerobes, indicate that the in vitro susceptibility of borreliae probably does not resemble that of common Gram-negative bacteria [42,52,58,81]. The naturally occurring DNA-gyrase of *B. burgdorferi* s.l. and the homologous C-terminal domain of *Escherichia coli* GyrA are biochemically distinct, sharing only 24% identity at the amino acid level [98]. The GyrA C-terminal domain of *E. coli* is acidic with a predicted isoelectric point of 4.0, whereas the naturally occurring 34 kDa protein in *B. burgdorferi* s.l. is basic, with a predicted isoelectric point of 9.1 [98]. These differences may explain the lower activity of class I and II quinolones against borreliae in comparison to common Gram-negative bacteria, such as *E. coli*.

### 5.6. Aminoglycosides, Glycopeptides, Streptogramins, Fusidic Acid and Nalidixic Acid

When tested against 11 *B. burgdorferi* s.l. isolates, typical aminoglycoside derivatives such as tobramycin, amikacin, apramycin and ribostamycin showed no or no significant in vitro activity. Spectinomycin was the anomaly with MIC_50_ and MIC_90_ of 0.5 and 2 µg/mL respectively; the MIC_90_ of all other compounds was ≥32 µg/mL. Similarly, borreliae were fully resistant to fusidic acid and nalidixic acid, with MIC_90_ values > 4 and >256 µg/mL, respectively [17,52]. Interestingly, vancomycin (MIC_90_: 1 µg/mL), daptomycin and linezolid, which usually do not have in vitro activity against common Gram-negative bacteria, displayed a significant antibiotic effect against *B. burgdorferi* s.l. [42,65,99]. The same is true for streptogramins such as quinupristin/dalfopristin (MIC_90_: 0.125 µ/mL) but not for teicoplanin (MIC_90_: >8 µg/mL) [17,52]. Concerning sulphonamides, dapsone, sulfachlorpyridazine and trimethoprim drugs showed low activity against the stationary phase of *B. burgdorferi* s.l., and sulfamethoxazole was the least active drug among them in vitro. It is worth noting that trimethoprim did not show synergy in the drug combinations with the three sulfadrugs. However, sulfadrugs and trimethoprim, when combined with other antibiotics such as doxycycline, ciprofloxacin and cefuroxime, were more active than the respective single drugs. Nevertheless, none of the sulfadrug combinations were as efficacious as daptomycin and they were unable to completely eradicate borreliae as stationary phase cells in vitro [100]. Hygromycin A, a known antimicrobial produced by *Streptomyces hygroscopicus*, targets ribosomes and is selectively taken up by *B. burgdorferi* s.l. Recently, it has also been found that hygromycin A accumulates in *B. burgdorferi* s.l. and can eradicate LB in mouse models. Moreover, it was less disruptive to the fecal microbiome than other clinically relevant antibiotics because of its selective uptake by borreliae [69].

Table 2 summarizes the current in vitro data on the susceptibility pattern of *B. burgdorferi* s.l. with regard to established and more recently introduced antimicrobial agents. The MIC_90_ values determined using micro- and macrodilution methods for traditional ß-lactams, carbapenems, macrolides, tetracyclines, quinolones and glycopeptids against borreliae are more or less in agreement with the MIC values provided by other investigators in the current literature [18,36,37,39,40,42,43,99]. Based on these data, mezlocillin, piperacillin, ceftriaxone, azithromycin, telithromycin and cethromycin appear to have the greatest in vitro activity on a µg/mL basis, exhibiting low MIC_90_ values ≤ 0.03 µg/mL and MBC_90_ values ≤ 2 µg/mL. For all of the other antimicrobial agents, the MIC_90_ values were found to be ≥0.06 and >2 µg/mL [17,52]. This is substantiated further by recent findings that azlocillin completely kills late log phase and 7 to 10-day-old spirochetes in the stationary growth phase, and that azlocillin and cefotaxime can efficaciously kill in vitro doxycycline-tolerant *B. burgdorferi* s.l. Moreover, a combination of azlocillin and cefotaxime completely killed doxycycline-tolerant spirochetes. When tested in vivo, azlocillin has shown good efficacy against *B. burgdorferi* s.l. in a mouse model [101]. These findings suggest that azlocillin, mezlocillin, and piperacillin may be more efficacious in treating *B. burgdorferi* s.l. than traditional penicillin. Future research on these penicillin derivatives and evaluation of their potential role in treating LB are warranted.

## 6. Differences in the Antimicrobial Susceptibility of *Borrelia burgdorferi* Genospecies

The heterogeneity of the antibiotic susceptibilities of *B. burgdorferi* s.l. isolates to antimicrobials has been debated in the scientific literature [17,102,103]. In order to demonstrate in vitro the possible differences in the MIC and MBC values for some targeted antimicrobial agents at the genospecies level, a variable number of isolates belonging to different genospecies have been investigated using varied parametric and non-parametric statistical approaches [36,51,55,58,59,65,83,104]. Several studies used the Kruskall-Wallis test in the genospecies-based statistical analysis of measured MIC values. They found slight but significant differences between the in vitro susceptibilities (MIC values) of the various genospecies to penicillin, amoxicillin, aztreonam and quinupristin/dalfopristin [51,55,65]. Similarly, careful evaluation of the MBC values for quinolones revealed that the MBC values of grepafloxacin, clinafloxacin, sitafloxacin and gemifloxacin in *B. burgdorferi* s.s. isolates were significantly higher than for *B. afzelii*, *B garinii*, *B. valaisiana* and *B. bissettiae* isolates (*p* < 0.05) after 72 h of incubation [58]. Other authors, however, found no significant differences in the susceptibilities of strains belonging to the different *Borrelia* genospecies that are pathogenic for humans [44]. This is most likely due to differences in study designs, the number of isolates used, and the test methodology. Time-kill experiments, however, found lower in vitro activity for some quinolones and ketolides in *B. burgdorferi* s.s. isolates than in the other *Borrelia* isolates investigated [52]. These data again point to a somewhat slower killing rate by these antimicrobial agents in some *Borrelia* isolates and to possible differences in the spirochete drug interactions with regard to the genospecies evaluated [58,59]. This could be of interest, as *B. burgdorferi* s.s. and *B. afzelii* (which represents the most frequently isolated human-pathogenic genospecies in Europe [105]) seem to be significantly less susceptible to some antimicrobial agents in vitro. In contrast, *B. garinii* isolates appear to be more sensitive to many antibiotics than isolates of the genospecies *B. afzelii*, *B. burgdorferi* s.s., *B. bissettiae*, and *B. valaisiana* [51,55,58,65]. These findings are in close agreement with experimental data published by Preac-Mursic et al. [75], Peter et al. [104] and Sicklinger et al. [83], demonstrating that *B. garinii* strains are more sensitive to the antimicrobial agents that are used therapeutically. Finally, differences in antibiotic susceptibility also exist within a single species [7,75]. As demonstrated earlier by Preac-Mursic et al. [75], individual bacteria within a wild-type population can differ slightly in their susceptibility to various antimicrobial agents. Nevertheless, the observed differences are largely marginal and of questionable clinical relevance for the treatment of LB, as they usually do not exceed the critical serum concentrations for most substances to become inefficacious [7,83,106]. Unfavorable pharmacodynamic and pharmacokinetic conditions in some LB patients infected with “resistant” strains may enable the survival of spirochetes at immunologically privileged sites, despite prolonged antibiotic therapy [52]. Moreover, it has been proposed that the more challenging long-term manifestations of LBsuch as ACA and LA, may be associated in part with more-resistant strains [17]. To date, however, the testing of first isolates and strains derived from patients with spirochetal persistence after treatment has been an absolute exception [17]. The recovery and comparison of such isolates is clearly required in order to substantiate these speculations [17,52,58].

## 7. Drawbacks and Challenges for Susceptibility Testing of Borreliae In Vitro

Taking into account the aforementioned technical limitations and drawbacks, as well as the lack of internationally accepted standardized methodology [17,36,40], current in vitro susceptibility data on MIC and MBC of antimicrobial agents for borreliae are far from consistent [17]. To date, no less than 14 MIC definitions and 11 MBC definitions (Table 1) have been proposed in the scientific literature with regard to susceptibility testing of borreliae [17,36,37,39,40,43,49,64,67,68]. In addition, testing of *B. burgdorferi* s.l. is significantly influenced by considerable differences in testing conditions, i.e., incubation periods (48 h–10 days), the variable density of the inoculum (10^4^ to ~ 10^7^/mL), the composition of the test medium [17,36,84,107,108], obvious interactions between the antimicrobial substance and the test medium, the reading mode, the criteria to correctly determine antibiotic-induced killing, and growth inhibition in vitro [36,40,64,107]. Such inconsistencies account for the wide variability of published MIC and MBC values. For example, when reviewing studies in the literature, the reported MIC of penicillin G varies from 0.003 to 8 µg/mL and the MBC varies from 0.05 to >50 µg/mL [17,38,51,64,109]. Similarly, for doxycycline, the MIC varies from 0.06 to 2 µg/mL and the MBC varies from 0.25 to 6.4 µg/mL [17,39,51,64,109]. The results obtained by these studies are also limited as only small numbers of isolates (two to 30 strains) were examined. In contrast, investigations into common rapid growers typically involve large numbers of clinical isolates [17,44,110]. This is additionally a constraint as susceptibility testing is mostly conducted on easily accessible, high-passage isolates from stock cultures known from the literature and is rarely performed on newly isolated low-passage strains, or on isolates directly obtained from patients showing resistance to treatment [51,53,81].

### Discrepancies in In Vitro and In Vivo Activities: Interactions of Antimicrobials with BSK Medium

The in vitro efficacy of many traditional antimicrobial agents against borreliae has not always correlated with clinical experience [17,18,19,20,21,42,61]. This observation is partly explained by experiments that point to possible interactions between antimicrobial agents and BSK medium, indicating that the chemical instability of some substances, and the side-effects resulting from enzymatic activity of BSK components, are capable of influencing the in vitro testing of borreliae during the lengthy incubation periods (several days to weeks) [17,36,40,52,84,108]. Some ß-lactams, such as penicillin G and amoxicillin, display only moderate to good in vitro activity against *B. burgdorferi* s.l. but are clinically very efficacious in most patients. This can be explained, on the one hand, by the fact that the activity of some ß-lactams, both in vitro and in vivo, is temperature-dependent. For example, the in vitro activity of penicillin against borreliae increases up to 16-fold after temperatures are raised from 36 °C to 38 °C [110]. On the other hand, for many ß-lactams, a possible loss of activity during incubation is due, in part, to their poor chemical stability [36,51,107,108]. For instance, a significant decrease in antimicrobial efficacy, ranging from 66.3% for mezlocillin to 85.9% for penicillin, was found in *Bacillus subtilis* bioassays after 72 h of incubation in BSK medium [36,40]. Similarly, the activity of trimethoprim is diminished in the presence of BSK medium because this medium contains thymidine and p-aminobenzoic acid. When the medium was modified by reducing the components causing trimethoprim inhibition, the borreliae were shown to be somewhat susceptible to this drugs in vitro [111]. Consequently, for most antimicrobial agents, a possible loss of activity during incubation is mostly attributable to their poor chemical stability or to side-effects resulting from the enzymatic activity of BSK components [55,84].

The opposite is true; however, for chloramphenicol as the serum esterase present in BSK medium can reconvert inactive diacetyl-chloramphenicol to the active form of the antibiotic. Thus, the in vitro findings measured in BSK medium do not accurately reflect the level of chloramphenicol activity against borreliae in vivo [84]. Remarkably, on a µg/mL basis, macrolides are also more efficacious against borreliae than tetracyclines when tested in BSK medium [51,55]. In contrast, the clinical administration of macrolides, such as erythromycin and roxithromycin, is frequently unsuccessful in treating LB or is followed by a clinical relapse after the conclusion of the treatment [7,15,16,61]. Similarly, high rates of treatment failure have been reported in patients with primary and secondary syphilis that have been treated with erythromycin [61,112]. The reasons for this known variability in the clinical efficacy of macrolides against spirochetes remain, in part, controversial and are a focus of ongoing scientific research. This is why it is so important to perform control experiments. For example, while testing macrolides and ketolides against borreliae, control experiments with a reference strain of a fastidious organism (e.g., *S. pneumoniae* ATCC 49619) revealed possible interactions of the BSK medium with these antimicrobials. It became obvious that the activity of most of the macrolide derivatives tested increased in BSK medium, as revealed by MIC values below the range published by the Clinical Laboratory Standards Institute (CLSI) for these agents against *S. pneumoniae* [59]. Possibly, the lower protein binding of macrolides in BSK-containing rabbit serum may contribute to the increased activity of the macrolides against borreliae in vitro [61]. Some authors, therefore, speculate that the known high variability of species-dependent protein binding of roxithromycin and erythromycin (ranging from 7% in rabbits to 86% in humans) may be the reason for the excellent in vitro activity of both drugs against borreliae. This contrasts sharply with the, sometimes, poor clinical performance in cases of human LB [7,61]. This is important as far as the “true MIC values” of the macrolide agents in vivo most likely need to be estimated as being at least one or two log_2_ unit dilutions higher than those measured in BSK medium [52].

Furthermore, the analysis of genes that encode ribosomal proteins in eight eubacterial species, including *B. burgdorferi* s.l., demonstrated that, although several 16s-RNA encoding genes considered to be of structural importance are conserved throughout the bacterial species, the degrees of sequence conservation can differ from one ribosomal protein gene to another [113]. Therefore, impact of this variation on aspects of pharmacokinetic variability and drug interactions must be taken into account. Typical factors that come into play are protein binding, plasma half-life, bioavailability, variable concentrations in various body compartments, and differences in the binding affinities of the traditional macrolides to the corresponding targets of the ribosome. These factors, alongside others, may be responsible for the striking difference between excellent in vitro activity and poor in vivo efficacy of these substances against *Borrelia* and *Treponema* [52]. Further experiments involving serum-depleted BSK medium or BSK medium that have been supplemented with human serum instead of rabbit serum are needed in order to gain insight into interactions of the test medium with the antimicrobial agents in general, and the quinolones, macrolides and ketolides in particular.

Clearly, more studies are needed to resolve the currently known discrepancies between the in vitro and in vivo activity of antimicrobial agents against the Lyme disease spirochete [52]. The variability of MIC data and the somewhat uncommon susceptibility pattern of borreliae may also result from differences in binding affinities of antimicrobial agents to the corresponding *Borrelia* target proteins (e.g., penicillin-binding proteins, ribosomes and DNA-topoisomerases) under in vitro testing conditions. Most importantly, some of the proven inconsistencies in the susceptibility pattern of borreliae are due to the fact that the unique cell envelope of these spirochetes clearly has features in common with both Gram-positive and Gram-negative bacteria [81].

## 8. Possible Mechanisms of Resistance of *Borrelia burgdorferi* s.l. In Vitro

### 8.1. Currently Known Phenotypic and Genotypic Mechanisms of Resistance

*Borrelia burgdorferi* s.l. is currently not known to have traditional antimicrobial resistance mechanisms and is generally susceptible to many antimicrobial agents in vitro [53,81]. In addition, humans represent a dead-end host and the further spread of whatever resistance to other strains is rather unlikely in this vector-borne organism, at least under natural real-life conditions outside the laboratory. However, a small number of studies have demonstrated the development of acquired resistance mechanisms in both laboratory and clinical settings. A development of resistance to erythromycin (100 to 500 µg/mL) has been reported in clinical isolates of *B. burgdorferi* s.l. [114]. A low-passage clinical isolate with an unusually high level of resistance to macrolides and lincosamides was phenotypically shown to have a modified ribosome structure when radio-labelled erythromycin was used. However, the genes encoding this resistance could not be clearly identified. The resistance determinant appeared to be encoded by mobile genetic elements because the phenotype was transferable to other bacterial species [114]. Based on conjugation frequency, the elements were thought to be constins or integrating conjugative elements.

Furthermore, a TolC-like efflux system, in which the outer membrane porin BesC plays an integral role, has been identified in *B. burgdorferi* s.l. This mechanism of antibiotic resistance results from the efflux of antibiotics from the spirochete through the use of a resistance-nodulation-division (RND) efflux pump called BesABC [115]. A *besC* knockout in a genetically modified *B. burgdorferi* s.s. strain was shown to result in an increased susceptibility to multiple classes of antibiotics used to treat LB. For tetracycline, an 8-fold decrease in the MIC_90_ value (0.31 µg/mL in the wild-type strain vs. 0.04 µg/mL in the *BesC* knockout strain) and a 15-fold decrease in the MBC value (2.5 µg/mL in the wild-type strain vs. 0.16 µg/mL in the *besC* knockout strain) was observed using standardized colorimetric susceptibility testing [115].

Resistance to coumermycin (due to mutations in the *gyrA* gene), to aminoglycosides (due to homologous mutations in the small ribosomal RNA subunit), and to fluoroquinolones (due to mutation in the *parC* gene) have been demonstrated following mutant selection in the laboratory [116,117]. Nonetheless, relatively little is known to date about the in vitro pharmacodynamic interactions between new fluoroquinolones and *Borrelia* spp. The observed increase in in vitro efficacy of some of the 4-quinolones against borreliae, as outlined above, is consistent with those of other investigators who demonstrated the activity of ciprofloxacin, moxifloxacin, sparfloxacin and the DNA-gyrase inhibitor coumermycin A1 against *B. burgdorferi* s.l. [40,96,97]. Interestingly, investigations into *S. pneumoniae* revealed that, in addition to gemifloxacin’s activity against the pneumococcal DNA-gyrase, the improved efficacy of the drug is probably related to its remarkably high affinity to topoisomerase IV [118]. Similarly, the activity of gemifloxacin, sitafloxacin and grepafloxacin against borreliae is approximately 10 to 100 times higher than that of the older 4-quinolones, but clearly lower than that of ceftriaxone, which served as a control substance as it is known to display a high rate of activity [58]. Furthermore, time-kill experiments indicate that gemifloxacin has a stronger cytotoxic effect on borreliae than ciprofloxacin [58]. Thus, it can be speculated that enhanced in vitro activity of gemifloxacin and sitafloxacin against borreliae may be due to a higher affinity to one of the *Borrelia* topoisomerases. This hypothesis is supported by recent experimental data demonstrating that mutations in *parC*, which encodes a subunit of *Borrelia* topoisomerase IV, were associated with a loss of susceptibility to sparfloxacin and moxifloxacin, but not ciprofloxacin [96]. Little or no bactericidal activity for ciprofloxacin and gemifloxacin occurs in the *Borrelia* isolates during the first 24 to 72 h of exposure, however, killing rates increase markedly after longer incubation periods [58]. These observations are similar to those obtained previously for *Enterococcus faecalis*. Here a much slower antibiotic killing is observed in comparison to *S. aureus* after exposure to ciprofloxacin and ofloxacin [119]. In borreliae, this finding may be due to the longer generation time required for the replication cycle and cell division. As such, detection of impaired plasmid relaxation and supercoiling in the *B. burgdorferi* s.s. strain B31, resulting from coumermycin A_1_ requires at least 2 h compared to only around 20 min for *E. coli* [97].

As outlined above, hygromycin A is a highly discerning antibiotic for spirochete infections such as LB and is selectively taken up through a nucleoside transporter found in spirochetal bacteria. However, systemic acquired resistance can occur after exposure to the antibiotic in vitro. Unter experimental conditions, two mutants—*B. turcica* KLEX1 and *B. burgdorferi* B31 KLEX2—were selected that showed stable hygromycin A resistance. Monitoring the transcriptome of cells treated with hygromycin A showed a 3.18-fold decrease in the expression of BmpD, a periplasmic substrate-binding protein of an ABC-type purine nucleoside transporter, followed by a 16-fold increase in the MIC of hygromycin A compared to the wild type [69].

### 8.2. Tolerance of Borrelia burgdorferi s.l. When Exposed to Antimicrobial Agents

Tolerance to metabolic, chemical and physical challenges, including antimicrobials, is a crucial—if not obligatory—phenotype of *B. burgdorferi* s.l. It is necessary in order to complete its enzootic cycle in mammalian and possibly avian reservoirs and in ticks. Several pathways and genes have been identified that could be involved in the generation of antimicrobial tolerance in *B. burgdorferi* s.l. These include the stringent response mediated by Rel and DksA, synthesis of the quorum sensing factor AI-2 mediated by LuxS, and the modulation of the levels of ATP and protein aggregation indirectly mediated by the GTPase CgtA (ObgE) [120]. Another factor that could produce antimicrobial tolerance in *B. burgdorferi* s.l. is a decrease in growth rate triggered by a scarcity of nutrients [120].

*Borrelia burgdorferi* s.l. can form antibiotic-tolerant persisters in the presence of microbiostatic drugs such as doxycycline [121]. It is currently unknown how this occurs. Some authors speculate that the multiple regulatory pathways involved in mediating this tolerance to antimicrobials and environmental stressors by persistence might include genes of the stringent (*rel* and *dksA*) and host adaptation (*rpoS*) responses, sugar metabolism (*glpD*), and polypeptide transporters (*opp*) [120]. When next-generation RNA sequencing was used on doxycycline-treated spirochetes and spirochetes treated following regrowth, and compared to untreated borreliae, genes upregulated in the treated *B. burgdorferi* s.l. included a number of Erp encoding genes and *rplU*,a gene coding for a 50S ribosomal protein. Genes associated with post-treatment regrowth included *bba74* (Oms28), *bba03*, several peptide ABC transporters, *ospA*, *ospB*, *ospC*, *dbpA* and *bba62* [121]. One of the reasons proposed by this study for the increased activity of tigecycline against borreliae compared to traditional tetracyclines may be the fact that tigecycline is obviously more resistant to efflux, as mediated by RND-type efflux systems, than are the traditional tetracyclines [57,122,123]. This parallels findings where tigecycline was shown to be more efficacious than traditional tetracyclines in many Gram-positive and Gram-negative bacteria, including multi-drug resistant pathogens with RND-efflux pumps [122]. Such RND-type efflux pumps are prevalent in a wide variety of rapid growers, where they appear to decrease the susceptibility of these organisms to antibiotic agents, such as tetracyclines [124].

*Borrelia burgdorferi* s.l. is pleomorphic and can generate viable but non-culturable bacteria which could also be a component in antimicrobial tolerance. The term “antimicrobial tolerance/persistence” is commonly used to describe the small fraction of single-cell heterogeneous antimicrobial-tolerant persister cells. The antimicrobial-tolerant persister phenotype is an epigenetic rather than a genotypic property; a culture of isolated single persister bacteria in fresh medium without an antimicrobial generates a newly heterogenous bacterial population that mainly contains susceptible cells and a small fraction of antimicrobial-tolerant cells with a new biphasic killing curve on re-exposure [120]. Several studies have confirmed the presence of antimicrobial-tolerant cells in *B. burgdorferi* cultures. Common to antimicrobial-tolerant persistent bacteria is an increase in the minimum time needed to kill 99.99% of the population (MDK99.99) as well as heterogeneity in cellular susceptibility in culture [120]. This was first suggested by the increased tolerance of stationary-phase borreliae to doxycycline, amoxicillin and nitrofurantoin, and by alterations in spirochete morphology, including the formation of round bodies [125,126]. Cultures exposed to doxycycline, amoxicillin or ceftriaxone displayed biphasic killing curves typical of cultures containing tolerant cells whose numbers increased during the stationary phase. The tolerance to these antimicrobials was not heritable [72,121]. The emergence of spirochetes tolerant to doxycycline in stationary-phase cultures was stochastic and depended on bacterial density [72,121]. Such putative antimicrobial-tolerant cells could be killed in vitro by daptomycin, carbomycin, cefoperazone, vancomycin, or clofazimine on their own, or through a combination of doxycycline, daptomycin, and cefoperazone [127,128]. It is not clear whether pulsed antimicrobial treatment, as suggested by some authors, is efficacious in reducing the number of antimicrobial-tolerant *B. burgdorferi* s.s. in these cultures, as different results have been apparently obtained with pulses of doxycycline and ceftriaxone [72,120,121].

### 8.3. Changes in Protein Expression in Borreliae Exposed to Antimicrobial Agents

**Figure 5:**
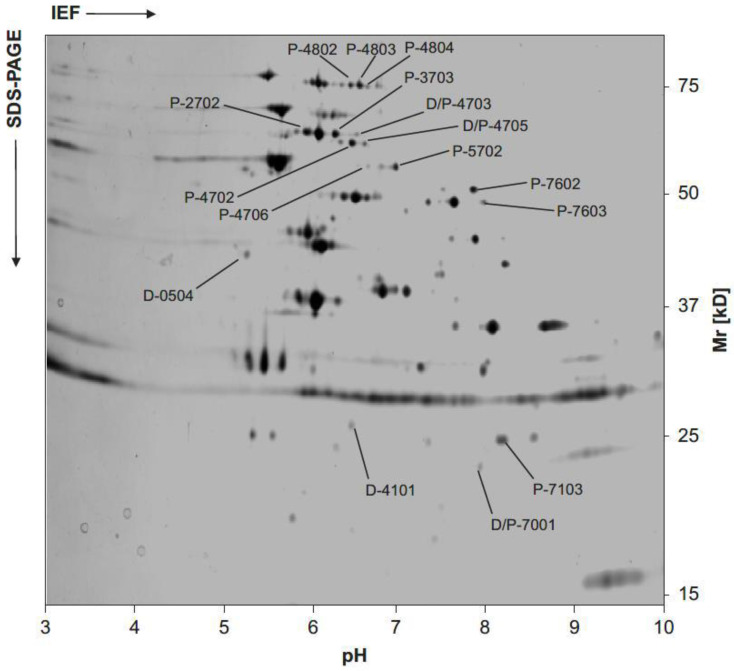
Representative two-dimensional gel analysis of *B. burgdorferi* s.s. isolate LW2 (adapted from [129]). Twenty micrograms of protein derived from a culture in the log-phase of growth were focused on a pH-gradient ranging between pH 3 and 10. After isoelectric focusing (IEF), the sample was separated through a 10% Tris/tricine SDS-PAGE and subsequently silver stained. Location and molecular mass of differentially expressed proteins in samples of borreliae exposed to penicillin G and doxycycline at concentrations around the MIC are indicated by straight lines. Such protein spots were then cored from preparative gels and further analyzed by MALDI-TOF MS (see Table 3). The positions of molecular mass standard in kDa are indicated on the right. The linear pH gradient ranging from pH 3 to 10 is indicated at the bottom of the photograph.

Proteomics, a snapshot-like analysis of a certain protein composition that is regarded as representative of the functional status of a defined biological compartment, is a growing field of research that encompasses environmental factors [52,129]. Here, proteins, the acting macromolecules in bacterial cells, can serve as crucial targets to better understand infectious diseases [130]. Currently, two-dimensional electrophoresis (2-DE) is the only method available for separating proteins from complex mixtures, such as cells or tissues. The approach with the highest resolution separates up to 10,000 protein species [131]. Therefore, it represents a very promising approach for providing additional insights into the physiological processes of bacterial cell metabolism. Additionally, morphological changes in *B. burgdorferi* s.l. in response to adverse environmental conditions have been reported to occur when cells are exposed to physiological stress. Typical stress factors known to induce such alterations are changes in pH, depletion of energy metabolism, serum starvation while growing in BSK medium, the presence of antimicrobial agents, and ageing [18,132,133,134,135]. These data suggest that borreliae can respond rapidly to altered environmental conditions through variable protein expression [135]. Exposure of the pathogens to penicillin and doxycycline under defined test conditions revealed that fifteen protein spots in the samples treated with penicillin G were down-regulated (Figure 5; Table 3). Similarly, four protein spots were identified on the gels of the doxycycline-treated samples that were downregulated, as revealed by a ≥ 50% decrease in spot intensity in comparison to untreated controls [17,52]. In three spots, borreliae exposed to both penicillin and doxycycline at the MIC and at ½ the MIC led to a downregulation of protein synthesis. After 72 h, one additional protein could be identified that was strongly over-expressed after cultivation of borreliae in the presence of doxycycline at the MIC. It is interesting to note that, in addition to other proteins, the p66 protein, a pore-forming protein of *B. burgdorferi* s.l., is downregulated in the presence of penicillin G [17,52]. Porins are known to be vital for the influx of antimicrobial agents into the cytoplasm of Gram-negative bacteria [136]. In addition, the absence of porins is associated with resistance to ß-lactams, which occurs in porin-deficient mutants of Gram-negative bacteria with increased resistance to antimicrobial agents [137]. Accordingly, one is inclined to speculate that the downregulation of porins by borreliae in the presence of ß-lactams may be an adaptive process to hinder the influx of these drugs through the outer membrane into the periplasmic space, thereby lowering their inhibitory activity in peptidoglycan synthesis [17,52]. Interestingly, a significant upregulation of the triosephosphate isomerase, a key enzyme of glycolytic carbohydrate metabolism, was observed in the presence of doxycycline. This enzyme is also known to be variably expressed in the amastigote stage during the complex development cycle of *Leishmania* spp. [138]. In borreliae, its upregulation in the presence of a bacteriostatic antibiotic agent may, again, be interpreted as a counteracting initiation of increased metabolic activity due to an adaptive or evasive mechanism on the part of the pathogen being exposed to the drug [17,52]. Similarly, borreliae are known to react to serum starvation by a complex protein expression response involving more than 20 proteins [135]. Equally, *E. coli* reacts to fatty acid starvation of the culture medium by accumulating spot-dependent guanosine tetraphosphate (ppGpp) and inhibiting rRNA synthesis [139]. These findings suggest that, although *B. burgdorferi* s.l. has a small genome and extremely limited biosynthetic capabilities, these spirochetes can rapidly respond to antibiotic exposure within 72 h of incubation [17,52].

### 8.4. Evidence from Interactions between Borreliae and Antimicrobial Agents and Eukaryotic Cells

*Borrelia burgdorferi* s.l. can be recovered long after the initial infection, even from patients treated with antibiotics. This indicates that it can resist eradication by host defense mechanisms and antibiotics [52]. One study found that borreliae are protected from the lethal action of a 2-day exposure to 1 µg/mL of ceftriaxone (10–20 times the measured MBC) when in the presence of human foreskin fibroblasts [140]. In the absence of fibroblasts, the study found that the spirochetes did not survive and, similarly, borreliae were not protected from ceftriaxone by glutaraldehyde-fixed fibroblasts or fibroblast lysate. These findings suggest that living cells are necessary for protection. The ability of the organism to survive in the presence of fibroblasts was not related to its infectivity. Fibroblasts can protect borreliae exposed to ceftriaxone for at least 14 days. Mouse keratinocytes, HEp-2 cells, and Vero cells showed the same protective effect, however, Caco-2 cells did not [140]. This is consistent with the findings of Brouqui et al. and demonstrates that eucaryotic cells can protect *B. burgdorferi* s.l. from the action of penicillin and ceftriaxone, but not from the action of doxycycline and erythromycin [141]. The intracellular location of spirochetes observed in this study suggests that antibiotics that penetrate cells might have greater efficiency under such conditions. Doxycycline and erythromycin were more efficacious than penicillin or ceftriaxone in killing borreliae in vitro when the organisms were grown in the presence of eukaryotic cells [141]. Thus, several eukaryotic cell types seem to provide the borreliae with a protective environment and may contribute to its long-term survival, at least in vitro [140].

Bacterial persistence in the presence of antimicrobials, with no apparent increase in the MIC of the organism, appears to be a possible mechanism of antimicrobial resistance in borreliae [7,127]. *B. burgdorferi* s.l. can form both round body cells and biofilm-like structures in vitro, which appear more resistant to antimicrobial agents [142,143]. Consequently, the formation of drug-tolerant persister cells, which has been demonstrated in vitro, may be another explanation for the persistence of borreliae and a lack of responsiveness to antimicrobial activity in vitro [72]. These findings offer a possible explanation for why, under certain circumstances, individual LB patients suffer from relapses, or why borreliae can be re-cultured from some patients after having undergone appropriate administration of antibiotic treatment [7,60]. These observations point to the necessity to explore and develop assays capable of creating experimental in vitro conditions, which mimic more closely the in vivo situation of patients treated for LB.

## 9. Evidence from Isolates from Patients and Primates with Spirochetal Persistence

Treating LB with amoxicillin, cefuroxime, ceftriaxone and tetracycline is a common way to successfully resolve the infection [7,16,144,145]. However, similar to antibiotic treatment failures in patients with syphilis, another form of spirochetal infection [146], cases of treatment failure have been reported for almost every antimicrobial agent recommended for treating LB [7,18,19,20,21,22,82,147,148,149,150,151]. The occurrence of spirochetal persistence in EM patients has already been demonstrated in clinical studies [63]. Following these observations, additional experiments investigated the individual in vitro susceptibilities of 10 clinical isolates obtained from five EM patients before and after chemotherapy (Table 4) [7]. In this unique study, the total number of individuals with persistent infection after the conclusion of chemotherapy accounted for 1.7% of culture-confirmed EM patients (N = 1148) seen from 1995 to 2000 at the LB outpatient clinic at the University Clinic in Ljubljana, Slovenia [7]. The results of molecular typing clearly substantiate that all but one patient with a positive follow-up culture remained persistently infected with the same *Borrelia* genospecies at the same peripheral location for several weeks despite antibiotic treatment [7,60]. All isolates were then tested under standardized conditions using the previously described colorimetric MIC method, and MBC values were determined in modified BSK medium using conventional subculture approaches [7,17]. Overall, *B. garinii* isolates tended to be more susceptible than *B. afzelii* isolates that had higher MIC and MBC values [7]. Testing of clinical isolates obtained from the same patient before the start and after the conclusion of antimicrobial therapy for EM did not reveal clinically significant differences, i.e., no increase or decrease for ≥2 log_2_ unit dilutions in the median MICs and MBCs [7]. Consequently, despite persistence there was no evidence for acquired antimicrobial resistance in these clinical *B. burgdorferi* s.l. isolates re-cultured after antimicrobial chemotherapy [7].

These observations corroborate those of Bockenstedt et al., who used xenodiagnosis to demonstrate persistence of borreliae for up to 3 months in 4 out of 10 animals after prolonged therapy with doxycycline and ceftriaxone in a mouse models [153]. The results for EM patients treated with antibiotics suggest that the population of spirochetes detected after chemotherapy may differ genetically from the bacterial population that had initiated the infection. This is further supported by observations that the plasmid pattern can differ in isolates belonging to the same genospecies cultured from the patient’s EM site before and after chemotherapy [7]. These differences are likely to result from the selection of clones that are capable of persisting with the selection pressure rapidly resulting in the selection of clonal subtypes of the same or co-infecting genospecies [7]. Similarly, the persistence of group A streptococci and *Chlamydia* spp. after chemotherapy treatment of an infection has been observed but is not necessarily equivalent to clinical treatment failure or a relapse of symptoms [154,155]. These findings are in concordance with animal experiments, demonstrating the survival of infectious *Borrelia* isolates in antibiotically treated mice. Survival was correlated with genetic recombination and diminished levels or complete loss of lp25 and lp28-1, linear plasmids that are known to carry genes that are important for the infectiousness of borreliae. Such attenuated residual spirochetes were well adapted to persist in their hosts, but were no longer transmissible to new mammalian hosts [153]. In one patient, however, the plasmid pattern of the two subsequent *B. garinii* isolates did not change at all despite antibiotic treatment. Therefore, in some individuals, survival of small numbers of bacteria may result in persistent complaints [7,60].

In a primate model of LB [156], in which antimicrobial agents were administered 4 to 6 months after infection, antimicrobial-tolerant persister cells could be identified/isolated following treatment, demonstrating that *B. burgdorferi* s.s. can withstand antimicrobial treatment administered post dissemination. In addition, spirochetes were recovered by xenodiagnosis in two of the three treated animals and a few slow-growing organisms were recovered by culture from each animal. Furthermore, *ospA* transcripts were detected in culture pellets from animal tissues [156]. In a follow-up study of *Rhesus macaques*, morphologically intact spirochetes could be observed in several organs of treated animals and adjacent to a peripheral nerve of an untreated animal. Antigen staining of probable spirochete cross-sections was also observed in the heart, skeletal muscle, and near peripheral nerves of treated and untreated animals. These findings support the notion that ongoing symptoms in some LB patients following treatment may be attributable to residual inflammation in and around tissues that harbor a low burden of persistent host-adapted spirochetes and/or residual antigens [157]. The exact mechanisms of such spirochetal persistence have not yet been identified, but eradication of persister cells has been achieved at least in vitro by pulse dosing with third-generation cephalosporins or daptomycin combinations with doxycycline and/or cephalosporins [127].

In terms of antimicrobial resistance, there is also circumstantial evidence for the development of acquired antimicrobial resistance in *B. burgdorferi* s.l. against a few antimicrobial agents at least under laboratory conditions in vitro. It has been speculated that erythromycin resistance may develop in *Borrelia* isolates from LB patients who have been pre-exposed to erythromycin. This is based on the occurrence of resistant subpopulations in vitro, even though the authors of that study were unable to provide a scientifically sound explanation of the underlying mechanism [114]. The possible occurrence of acquired resistance in borreliae is also supported by the generation of mutants resistant to quinolones and aminoglycosides after the continuous culture in the presence of subinhibitory concentrations of these drugs in vitro [96,158]. Similarly, mutations in the encoding 23S-rRNA gene of *Treponema pallidum* have been found to confer functional resistance to azithromycin in patients with clinical treatment failure after taking this drug to treat a syphilis infection [159]. The findings in a clinical *B. garinii* isolate cultured from an EM patient after azithromycin treatment, however, did not support increasing resistance after exposure to this agent [7]. Instead, the findings are similar to those of Pfister et al. [20] and Hansen et al. [61] in relapsed patients with early LB. This shows that isolates cultured after the conclusion of roxithromycin and ceftriaxone therapy remain fully susceptible to these agents in vitro. The absence of confirmed acquired resistance in organisms isolated from antibiotically treated patients, however, does not rule out phenotypic resistance mechanisms similar to those assumed to cause relapse in syphilis and leptospirosis patients [146,149]. Likewise, for another slow-growing organism, *Mycobacterium tuberculosis*, the Cornell model of latent tuberculosis, in which mice infected with *M. tuberculosis* are treated with antibiotics [isoniazid and pyrazinamide], initially produces no detectable bacilli by organ culture. However, reactivation of infection during this culture-negative state obviously occurs spontaneously or following immune suppression after the conclusion of treatment. Such reactivation is similarly documented for LB and syphilis in the literature [18,20,75,146,160].

In addition, *B. burgdorferi* s.l. has been observed in cardiac myocytes of experimentally infected mice and in dermal macrophages in vivo [161,162]. Some authors have therefore speculated that *B. burgdorferi* s.l. overcomes the presence of ß-lactams by transitioning into a physiologically dormant state [75,134]. As outlined above, the protective effects of eukaryotic cells, as demonstrated in a study by Brouqui et al. [141], and a dormant state or temporary intracellular residence would offer protection for borreliae from both the host’s defense and antimicrobial agents. Interestingly, *B. burgdorferi* s.l. isolates can indeed adhere to, invade and survive in human endothelial cells [163,164]. These phenomena confirm, at the very least, the protective effects of mammalian cells on spirochetes in vitro. Very few studies exist, however, that, under standardized experimental conditions, have investigated the activity of common antimicrobial agents against borreliae in the presence of eukaryotic cells. Such an experimental design is much closer to the in vivo situation in individuals infected with *B. burgdorferi* s.l. and may provide important insights into possible evasion strategies of spirochetes—organisms that, despite their mainly extracellular residence are known for their close and sophisticated interactions with the various tissues of their hosts during the infection.

## 10. Summary and Conclusions

Over the last two decades, relatively little progress has been made in identifying possible mechanisms of persistence and antibiotic resistance for *B. burgdorferi* s.l., as has been the case for other bacterial pathogens. We still have many lessons to learn about the interactions between antimicrobial agents and *B. burgdorferi* s.l., as well as the potential mechanisms of spirochetes may have devised to overcome the presence of antimicrobial agents and host immunity in vivo. Recent studies provide evidence for the development of acquired in vitro resistance of *B. burgdorferi* s.l. against selected aminoglycosides, hygromycin A, and quinolones after prolonged exposure to subinhibitory concentrations of these drugs during in vitro culture. To date, however, there is no scientific evidence for clinically relevant acquired antimicrobial resistance against drugs that are commonly used for the therapy of *B. burgdorferi* s.l. infection as a cause of culture-confirmed persistence of borreliae in LB patients. The public health impact on patients with the persistence of borreliae after antimicrobial therapy is difficult to assess. Extrapolating the above-mentioned numbers from clinical investigations, a persistence rate of 1.5% in EM patients [7] and an annual number of >200,000 cases of LB in Germany alone [11] would add up to ~3000 patients with possible persistence. Such a spirochetal persistence, however, would go unnoted as regular follow-up cultures are not performed in these individuals under routine clinical conditions. In order to address the issues of treatment resistance and persistence in the presence of antimicrobial agents, further investigations need to be conducted on more clinical isolates obtained from LB patients after chemotherapy. This task, however, remains challenging, as the overall culture detection rate of the pathogen in clinical specimens obtained from cutaneous lesions usually does not exceed 40 to 70% [105,151,165,166,167]. Moreover, culture positivity even falls to <1% in cases with Lyme arthritis and 20% in cases with neuroborreliosis [105], and cultivation is rarely successful after antimicrobial therapy [7,152]. During the last decade, novel antimicrobial agents have been introduced and may become therapeutic alternatives for a variety of human infections. In addition to ongoing clinical trials on the optimum treatment regimen for LB, further basic research is urgently needed in order to better understand possible genetic or phenotypic mechanisms of persistence in *Borrelia* spp. In light of the excess morbidity and financial burden resulting from the healthcare challenges of LB patients, these issues contribute to critically important ongoing major public health issues [17,61,114]. Therefore, additional investigations involving clinical first isolates alongside in vitro and in vivo experiments are clearly necessary, not only to determine more tailored treatment strategies for patients with LB [17,52] but also to achieve in a better understanding of the critical interactions between spirochetes and antimicrobial agents [60].

## Figures and Tables

**Table 1:** Representative examples of MIC and MBC definitions for in vitro testing of *B. burgdorferi* s.l.

Reference	MIC ^a^ Definition	MBC ^b^ Definition	Inoculum	Exposure to Antimicrobial Agent	Method
Agger et al., 1992 [41]	The lowest concentration of antibiotic without motile borreliae (microscopic reading mode)	The lowest concentration of antibiotic without growth of borreliae after 7 days of subculture in a liquid medium (microscopic reading mode)	10^5^/mL	7 d	Macrodilution
Alder et al., 1993 [66]	The lowest dilution of antibiotics showed less than 5% vital borreliae (microscopic reading mode)	Not performed	5 × 10^6^/mL	48 h	Macrodilution
Berger et al., 1985 [67]	The lowest concentration of antibiotic showed no greater number of motile borreliae than the initial inoculum and more than 90% motility of cells (microscopic reading mode)	The lowest concentration of antibiotic without growth of borreliae 11 days of subculture in liquid medium (microscopic reading mode)	10^5^/mL	72 h	Macrodilution
Dever et al., 1992 [40]	The lowest concentration of antibiotic without macroscopic growth (pellet) or indicator color shift (macroscopic reading mode)	The lowest concentration of antibiotic with a 99.9% reduction of initial inoculum in subculture after 10–12 days (subsurface plating)	10^5^/mL	72 h	Microdilution
Dever et al., 1992 [40]	The lowest concentration of antibiotic without macroscopic growth (pellet) or indicator color shift (macroscopic reading mode)	The lowest concentration of antibiotic without growth of borreliae in a liquid medium after 10–12 days (macroscopic reading mode)	10^5^/mL	72 h	Macrodilution
Hansen et al., 1992 [61]	Not performed	The lowest concentration of antibiotic with complete growth inhibition of borreliae in subculture after 7 days (microscopic reading mode)	10^5^/mL	7 d	Macrodilution
Hunfeld et al., 2000 [51]	The lowest concentration of antibiotic without detectable indicator color shift (photometric reading mode)	The lowest concentration of antibiotic without re-growth of borreliae after 3 weeks of subculture in a liquid medium (microscopic reading mode)	2.5 × 10^7^/mL	72 h	Microdilution
Koetsveld et.al., 2017 [48]	The lowest concentration of antibiotic without detectable indicator color shift (photometric reading mode)	The lowest concentration of antibiotic without re-growth of borreliae after 3 weeks of subculture in a liquid medium (microscopic reading mode)	5 × 10^7^/mL	72 h	Microdilution
Johnson et al., 1984 [68]	The lowest concentration of antibiotic showed no greater number of motile borreliae than the initial inoculum (microscopic reading mode) and no sediment	Not performed	10^5^/mL	5 d	Macrodilution
Luft et al., 1988 [64]	The lowest concentration of antibiotic shows no greater number of motile borreliae than in the initial inoculum (microscopic reading mode)	The lowest concentration of antibiotic without growth of borreliae after 3 weeks of subculture in a liquid medium (microscopic reading mode)	2 × 10^5^/mL	72 h	Macrodilution
Leimer et al., 2021 [69]	The lowest concentration of antibiotics that inhibited growth	The lowest concentration of antibiotic with 99.9% reduction of initial inoculum in subculture after 20 days (semi-agar plates)	10^6^/mL	7 d	Microdilution
Morsehd et al., 1993 [70]	The lowest concentration of antibiotic with complete growth inhibition (microscopic reading mode)	Not performed	10^4^/mL	8–10 d	Macrodilution
Mursic et al., 1987 [38]	The lowest concentration of antibiotic without detectable re-culturable organisms (microscopic reading mode)	Not performed	10^5^/mL	5 d	Macrodilution
Stiernstedt et al., 1999 [71]	The lowest dilution of antibiotic shows the same number of motile borreliae as the initial inoculum (microscopic reading mode)	The lowest concentration of antibiotic without re-growth of borreliae after 2 weeks of subculture in liquid medium (microscopic reading mode)	10^6^/mL	7 d	Dialysis culture
Sharma B et al., 2015 [72]	The lowest concentration of antibiotics that inhibited growth	The lowest concentration of antibiotic with 99.9% reduction of initial inoculum in subculture after 20 days (semi-agar plates)	10^6^/mL	72 h	Microdilution

^a^ MIC minimal inhibitory concentration, ^b^ MBC minimal borreliacidal concentration.

**Table 2:** In vitro activity of 50 antibiotics (grouped according to the corresponding class of antimicrobials) against *B. burgdorferi* s.l. (updated and modified from [17,52]).

Antimicrobials	Colorimetric MIC_90_ ^a^ (µg/mL)	MIC Range (µg/mL) (Adopted from the Literature)	MBC_90_^b^ (µg/mL) (100% Killing after 72 h)	MBC Range (µg/mL) (Adopted from the Literature)
**ß-lactames**				
Penicillin G	1	0.03–8	16	0.05–>50
Amoxycillin	1	0.03–2	32	<0.03–3.2
Piperacillin	0.06	<0.06–0.125	2	1.3–2.6
Mezlocillin	≤0.06	<0.06–1	2	0.125–2
Azlocillin	0.125	0.125	4	N.A.
Aztreonam	>64	2–>64	>64	64–>256
Cefaclor	16	16–128	>64	64–128
Loracarbef	32	4–32	>128	128–>128
Ceftibuten	32	4–32	>128	128–>128
Cefixime	1	0.25–4	32	0.8–32
Cefuroxime Cefpodoxim	0.25 4	0.06–0.5 1–8	16 32	0.25–>16 32–>32
Cefotaxime	0.125	0.01–1	8	0.02–0.25
Ceftriaxone	0.03	<0.01–0.125	2	0.02–3.81
Sulbactam	64	0.125–256	>64	>64
**Carbapenems**				
Meropenem	0.25	0.012–0.5	16	0.5–32
Faropenem Imipenem Ertapenem	4 0.5 0.125	0.03–8 0.06–0.5 0.015–0.125	64 32 4	8–>64 16–32 0.5–4
**Glycopeptides**				
Vancomycin	1	0.25–2	16	2–32
Teicoplanin **Lipopeptides**	>8	2–>8	>8	>8
Daptomycin	N.A.	0.25–32	>32	>32
**Tetracyclines**				
Tetracycline	0.25	0.01–20	N.A.	0.8–4.1
Minocycline	0.25	0.03–1	5.8 (mean MBC)	3–8
Doxycycline	0.25	0.06–2	N.A.	0.25–6.4
Tigecycline	>0.016	<0.016	0.25	0.25–1
**Macrolides**				
Erythromycin	0.062	≤0.007–1	>0.5	0.05–2.17
Roxithromycin	0.062	0.015–0.12	>0.5	0.0125–1.8
Azithromycin	0.015	0.003–0.03	0.5	0.007–0.5
**Streptogramins**				
Quinopristin/ Dalfopristin	0.125	<0.015–0.25	8	2–8
**Ketolides**				
Telithromycin	0.0078	<0.0002–0.0078	0.25	0.0156–0.5
Cethromycin	0.0019	<0.0002–0.0078	0.125	0.0156–0.25
ABT-773	>0.0020	>0.0020	0.35	0.02–1
**Cinnamic acids**				
Hygromycin A	N.A.	0.25	1	N.A.
**Aminoglycosides**				
Tobramycin	64	8–64	>64	N.A.
Amikacin	128	32–>128	>128	>128
Ribostamycin	>32	8–64	>32	4–>32
Spectinomycin	2	0.25–2	N.A	N.A.
**Quinolones**				
Nalidixic acid	256	128–>512	>512	≥512
Norfloxacin	8	1–16	>64	8–>64
Ofloxacin	8	1–16	>16	8–>16
Ciprofloxacin	2	0.25–8	>16	4–>16
Sparfloxacin Grepafloxacin Gatifloxacin Sitafloxacin	1 0.5 1 0.5	0.06–8 0.6–2 0.25–2 0.6–4	>16 16 >16 >16	2–>16 2–>16 4–>16 1–>16
Gemifloxacin	0.12	0.03–0.25	8	0.25–16
**Others**				
Fusidic acid	>4	>4	>4	>4
Chloramphenicol Linezolid Sulfamethoxazol	N.A. N.A. N.A.	1.25–2 >2 8	N.A. >2 >256	N.A. N.A. 32–>256.

**^a^** MIC_90_, MIC at which 90% of the isolates are inhibited. Colorimetric MIC were determined in *B. burgdorferi* s.l. isolates under standardized conditions. **^b^** MBC_90_, MBC at which 90% of the isolates showed 100% killing of the final inoculum after 72 h under standardized conditions. N.A., not available.

**Table 3:** Protein spots and proteins showing up- or down-regulation after exposure to *B. burgdorferi* s.s. strain LW2 to increasing concentrations of penicillin G, doxycycline, or both (mod. from [129]).

Spot	*p*I	Mol. Mass (kDa)	Up- or Down	Mol. Mass (kDa)	Identified Protein
	(Est. from Gel)	(Est. from Gel)	Regulation	(From Database)	(By MALDI-TOF MS)
P-2702	5.9	65	d	86.13	Membrane-associated protein
					P66 (BB0603)
P-3703	6.2	65	d	68.13	Membrane-associated protein
					p66 (BB0603)
P-4702	6.4	63	d	63.496	Ribosomal protein S1 (RpsA)
					(BB0127)
P-4706	6.7	63	d	NA	No match found
P-4802	6.4	75	d	NA	No match found
P-4803	6.5	75	d	80.298	Polyribonucleotide
					nucleotidyltransferase (PnpA)
					(BB0805)
P-4804	6.6	75	d	NA	No match found
P-5702	7	57	d	50.061	Pyrophosphate-fructose-6-
					phosphate-1-phosphortransferase (Pfp)
					(BB0727)
P-7103	8.1	25	d	30.417	Predicted coding region
					(BB0238)
P-7602	7.8	50	d	55.856	Glycerol kinase (GlpK)
					(BB0241)
P-7603	7.9	49	d	53.227	Pyruvate kinase (Pyk)
					(BB0348)
D/P-4703	6.5	65	d	NA	No match found
D/P-4705	6.6	63	d	NA	No match found
D/P-7001	7.9	23	d	26.569	Pfs protein (Pfs-1)
					(BB0375)
D-0504	5.2	42	d	42.372	Cell division protein (FtsZ)
					(BB0229)
D-4101	6.4	26	u	27.984	Triosephosphate isomerase

Proteins were identified by MALDI-TOF MS after preparative isolation from 2-DE gels. *p*I: isoelectric point; P: proteins affected by penicillin G exposure; D: proteins affected by doxycycline exposure; NA: not available. d: down-regulated; u: up-regulated.

**Table 4:** Clinical information and laboratory data on five patients with EM and a culture-confirmed persistent *B. burgdorferi* s.l infection after antimicrobial chemotherapy (modified from [152]).

	EM Patients				
**Clinical information**	1	2	3	4	5
Sex	F	F	F	F	M
Age (yrs.)	43	38	53	68	36
Symptoms at first visit ^a^	MEM	EM	EM	EM, L, PFP	EM, L
Recognition of tick bite	Yes	Yes	No	No	Yes
Size of EM (cm)	7 × 2; 6 × 8	10 × 16	7 × 11	9 × 12	7 × 12
Systemic complaints ^b^	None	None	Yes	None	None
Initial treatment Duration (d)	Ceftriaxone 1 × 2 g i.v. 14	Amoxicillin 3 × 500 mg p.o. 14	Cefuroxime 2 × 500 mg p.o. 14	Cefuroxime 2 × 500 mg p.o. 14	Azithromycin 2 × 500 mg p.o. 1 1 × 500 mg p.o. 4
IgM-IFT (titre) ^c^	256	128	Neg.	Neg.	Neg.
IgG-IFT (titre) ^c^	128	128	Neg.	Neg.	Neg.
Initial culture	*B. afzelii*	*B. garinii*	*B. afzelii*	*B. afzelii*	*B. afzelii*
MIC/MBC ^d^	0.0156/0.25	0.0312/0.5	0.0625/8	0.0625/8	0.0039/0.125
Follow-up culture	*B. afzelii*	*B. garinii*	*B. afzelii*	*B. afzelii*	*B. garinii*
MIC/MBC ^d^	0.0312/0.5	0.0625/1	0.125/16	0.0625/8	0.0019/0.125

^a^ EM: erythema migrans, MEM: multiple erythema migrans, PFP: peripheral facial palsy, L: lymphocytoma. ^b^ Systemic complaints: fever, headache, myalgia, ^c^ No significant titer change at second biopsy, ^d^ MIC/MBC (µg/mL) of antimicrobial used for specific therapy in the individual patients.
